# An in vitro evaluation of antimicrobial activity of a fast-setting endodontic material

**DOI:** 10.1038/s41598-022-20454-7

**Published:** 2022-09-26

**Authors:** Mengzhen Ji, Yaqi Chi, Ye Wang, Kaixin Xiong, Xuan Chen, Ling Zou

**Affiliations:** State Key Laboratory of Oral Diseases, Sichuan University, National Clinical Research Center for Oral Diseases, Department of Endodontics, West China Hospital of Stomatology, Sichuan University, Chengdu, 610041 China

**Keywords:** Antimicrobials, Bacteria, Pulpitis

## Abstract

The aim of this study is to evaluate the antimicrobial activity of the fast-setting iRoot Fast Set Root Repair Material (iRoot FS), Mineral trioxide aggregate (MTA) and Biodentine. The materials were freshly mixed or set for 1 and 7 days to conduct the agar diffusion test, direct contact test and carry-over effect test against *E. faecalis* and *P. gingivalis*, and the pH values were also measured. The data were analyzed by an analysis of variance and one-way ANOVA or Dunnett’s T3 test, and the Tukey’s post hoc test for multiple comparisons (α = 0.05). In the direct contact test, all three materials showed good antibacterial activity after setting for 20 min. The antibacterial properties of the three materials decreased with the increase of setting time (*p* < 0.05). The suspension of all the three materials showed high pH values (11–12) and no significant difference was observed (*p* > 0.05). With the extension of setting time, the pH of iRoot FS and Biodentine slightly decreased (*p* < 0.05). Fresh iRoot FS, Biodentine, and MTA killed *E. faecalis* and *P. gingivalis* effectively, but their antimicrobial effect decreased after 24 h, and distinctly decreased after 7 days after mixing. iRoot FS, Biodentine, and MTA showed a tendency of alkalinity during this 7-day experiment.

## Introduction

Endodontic surgery is an option to preserve teeth suffering from non-healing apical periodontitis when root canal retreatment has failed or is not possible^1^. An ideal root-end filling materials should possess certain characteristics, including biocompatibility, sealing ability, dimensional stability and low solubility^2^. Also, an ideal root-end filling material should possess antimicrobial activity to inhibit bacteria growth to prevent endodontic surgical failure caused by further microleakage^3^.

Mineral trioxide aggregate (MTA) is known as the “gold standard” for root-end filling material for its strong clinical performance^4^. In recent years, several kinds of materials went to the open-market^2^. Biodentine is a new version of calcium-silicate based inorganic cement and is commercially claimed to be a ‘bioactive dentine substitute’^5^. Further, a novel premixed calcium phosphate silicate cement, iRoot Fast Set Root Repair Material (iRoot FS, Innovative Bioceramix) was introduced to the market recently and has been used as a permanent root canal repair material in endodontic treatments and apical surgery^6^.

Previous study results were inconsistent in terms of measuring the antimicrobial activity of the retrograde filling materials used. MTA products were investigated in many articles through inconsistent methods^7–9^, There were also a few studies conducted on Biodentine^10,11^ , and none was found for iRoot FS. Therefore, the aim of this study was to evaluate the antimicrobial activity of these three retrograde filling materials: MTA, Biodentine, and iRoot FS.

## Materials and methods

### Specimen preparation

Five microgram of the ProRoot MTA (Dentsply, York County, PA, USA), Biodentine™ (Septodont, Saint Maur des Fosses, France) and iRoot FS (Innovative Bioceramix, BC, Canada) were prepared according to the manufacturers’ instructions and placed on 5 mm diameter sterile filter papers. The test samples were divided into three groups as described by Damlar et al.^3^. Briefly, samples tested at 20 min after mixing were designated as ‘fresh samples’, those tested on the first day after mixing were designated as ‘1-day samples’, and those tested on the seventh day after mixing were designated ‘7-day samples’. All the materials were allowed to set in a 100% moist atmosphere at 37 °C before experimenting. For the control groups, 5 mm diameter sterile filter papers were immersed in 0.12% chlorhexidine (CHX) and sterile saline respectively for 5 s before each test.

### Bacterial strains and culture conditions

Oral microorganism strains, *E. faecalis* (ATCC 19433) and *P. gingivalis* (ATCC 33277) were obtained from State Key Laboratory of Oral Diseases, Sichuan University, Chengdu, China. *E. faecalis* were cultivated in brain heart infusion broth (BHI broth, Becton, Dickinson and Company, US) and BHI agar plate, while *P. gingivalis* were cultivated in BHI broth and blood agar plate supplemented with 0.0005% hemin, 0.0001% vitamin K. Both strains were incubated anaerobically (N_2_ 80%; H_2_ 10%; CO_2_ 10%) at 37 °C.

### Agar diffusion test

Bacterial suspension was prepared for each bacterial strain and the turbidity was adjusted to 0.1 OD, which corresponds to approximately 10^8^ colony-forming units (CFU)/mL. Then 100 μL of *P. gingivalis* suspension was streaked on blood agar plates, while *E. faecalis* was streaked on BHI agar plates. A sterile scratcher was used to inoculate the bacterial suspension onto the agar plate to achieve a lawn of growth. The plates were dried for 5 s in room temperature before the filter papers coated with the materials, sterile saline or CHX were placed on each plate. The plates were cultured anaerobically (N_2_ 80%; H_2_ 10%; CO_2_ 10%) at 37 °C for 48 h before the diameter of the halo formed around the materials (inhibition zone) was observed. The tests were conducted in triplicate.

### Direct contact test (DCT)

The filter papers coated with the materials or sterile saline were placed at the bottom of 96-well plates, followed by 200 μL of the bacteria suspension (10^7^ CFU/mL) being added in each well in direct contact with the materials. After being cultured at 37 °C anaerobically for 1 h, the bacteria suspension transferred from each well were serially diluted. The survival of the microorganisms was determined by culturing 100 µL aliquots on BHI agar plates after they were serially diluted 10^3^–10^5^ fold. Then the colonies on the plates were counted and the CFU/mL value was calculated. The loss of viability was calculated by the following formula: loss of viability = (CFU control–CFU sample)/CFU control. The tests were conducted in triplicate.

### Carry-over effect test

The carry-over effects of the retrograde filling materials were assessed with procedures described by Ozcan et al.^12^ with some modifications. The filter papers coated with the materials or sterile saline were placed at the bottom of 96-well plates, and sterile saline (20 μL) was placed in direct contact with the materials. After incubation at 37 °C for 1 h, 230 μL of culture broth was added to each well. After mixing gently with a pipette, 20 μL of the broth was transferred to a tube containing 960 μL of culture broth. Then 20 μL of the bacteria suspension (1.5 × 10^8^ CFU/ml) was added to the tube. Ten-fold serial dilutions were prepared and plated onto BHI agar plates for colony forming. After incubation at 37 °C for 48 h, survival of the bacteria was compared between experimental groups and control group to investigate the antimicrobial activity of the materials. The tests were conducted in triplicate.

### The pH value measurement

For the pH value measurement, 25 mg of each endodontic material were mixed and evenly spread on the bottom of the 24-well plate and were allowed to set in a 100% moist atmosphere at 37 °C before experimenting, then 1 ml of distilled water (pH = 7.4) were added to each well after 20 min, 1 day and 7 days, respectively. After 1 h, the solution was drawn from the wells and centrifuged at 10,000 rpm for 10 min, and the pH measurement was performed with a Five Easy PluspHFEP20 pH meter (METTLER TOLEDO, Zurich, Switzerland).

### Statistical analysis

The data was subjected to a homogeneity test of variance using Levene’s test. Data with homogeneous variance were then statistically compared using one-way ANOVA, with the post hoc Tukey’s test. For data that showed heterogeneous variance, Dunnett’s T3 test was applied. Statistical analysis was performed using SPSS 21.0 (SPSS Inc., Chicago, IL, USA), and *p* < 0.05 was considered statistically significant.

## Results

### Antimicrobial activity

No inhibition zone was observed in the agar diffusion test except for the positive control.

**Figure 1:**
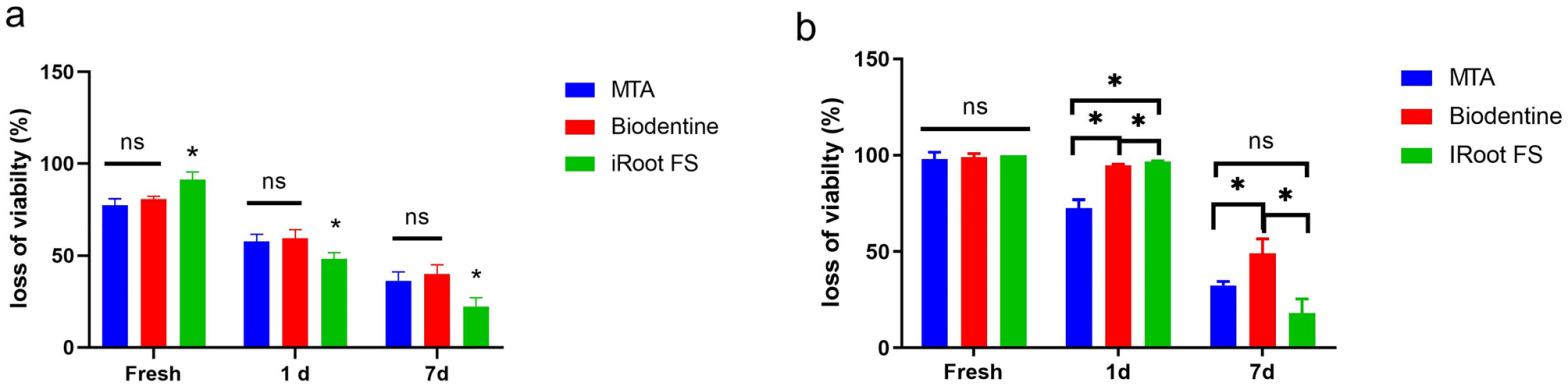
Outcome of direct contact test against (**a**) *E. faecalis* and (**b**) *P. gingivalis.*

The results of the DCT with *E. faecalis* and *P. gingivalis* are shown in Fig. 1a and b. The negative controls exhibited bacteria growth in all test periods. All three materials presented highest antimicrobial effect against *E. faecalis* and *P. gingivalis* when freshly mixed (*p* < 0.05). Fresh ProRootMTA, iRoot FS and Biodentine inhibited most *E. faecalis* (ProRootMTA 77.5%, iRoot FS 91.2%, Biodentine 80.7%), However, the antimicrobial activity of iRoot FS against *E. faecalis* were lower than the other two materials after setting for 1 or 7 days (*p* < 0.05). As for the antimicrobial activity against *P. gingivalis,* Fresh ProRootMTA and Biodentine inhibited almost all *P. gingivalis* (ProRootMTA 97.9%, Biodentine 98.9%) while iRoot FS inhibited the growth of all *P. gingivalis* (100%). However, no statistical significance was observed between the materials. iRoot FS and Biodentine produced almost complete inhibition after setting for 1 day, while the effect of MTA was relatively lower (*p* < 0.05). The 7-day samples of the three test materials showed significantly lower growth inhibition of *P. gingivalis* when compared with the other time interval groups (*p* < 0.05), while Biodentine showed relatively highest antimicrobial effect (*p* < 0.05).

**Figure 2:**
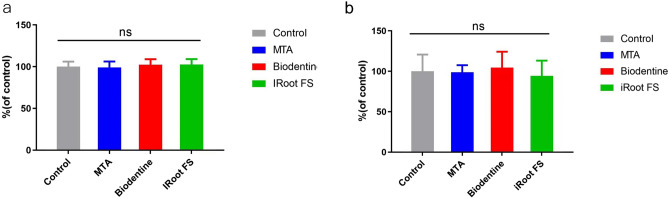
Outcome of carry-over effect test against (**a**) *E. faecalis* and (**b**) *P. gingivalis.*

Carry-over of the antimicrobial effect from the materials was not observed (*P* > 0.05) (Fig. 2).

### The pH values measurements

pH values of the leachate of the materials are shown in Table 1. All three endodontic materials showed significantly strong alkaline effect in all observed time intervals. No significant difference was noticed when freshly mixed among the materials, and iRoot FS presented lowest pH values after setting for 1 or 7 days (*P* < 0.05).

**Table 1:** The pH values of MTA, Biodentine, and iRoot FS leachates at different time intervals (mean ± S.D.;n = 3).

	MTA	Biodentine	iRoot FS
Fresh	11.85 (± 0.040)^A,a^	12.11 (± 0.012)^B,a^	11.87 (± 0.027)^A,a^
1d	12.01 (± 0.009)^A,b^	12.07 (± 0.026)^A,a^	11.81 (± 0.030)^B,a^
7d	11.46 (± 0.062)^A,c^	11.66 (± 0.038)^B,b^	11.00 (± 0.075)^C,b^

Superscript capital letters represent statistically significant differences in the same row, and superscript lowercases indicate statistically significant differences in the same column. The same letters indicate no significant differences among the compared groups (*p* > 0.05) and vice versa.

## Discussion

The DCT used in the present study is a quantitative and reproducible method to simulate the contact of the microorganism with retrograde filling materials^13^. This procedure allows us to assess the antimicrobial effect of test materials at different stages of the setting reaction, and also helps to determine whether the data reflect bactericidal, or just bacteriostatic effects, regardless of the diffusion rates of the active agents^14^. In 2009, Zhang et al. reported a modified DCT^15^, in which the suspension of MTA was obtained to contact the bacteria suspension. However, since the retrograde filling materials were in direct contact with the microorganisms inside the resected root canals^16^, the DCT applied in the present study might better mimic the clinical situation. The agar diffusion test (ADT) is another method to evaluate the antimicrobial activity of root-end filling materials^3^. Since the outcome of ADT depend on the material diffusibility in the medium^3^ , the solid root-end filling materials may not be diffusible, which could be a possible explanation for the negative outcome of ADT in the present study. Therefore, DCT seems more appropriate in evaluating the antimicrobial activity of solidified materials.

The results of DCT might be affected by carry-over effect of the materials since it can cause the growth inhibition of tested micro-organisms^17,18^. In the present study, no carry-over effect was observed. Since the root-end filling materials are insoluble, this result is expected. Therefore, the following discussion and conclusion are based on the results of DCT.

MTA was introduced innovatively as a root-filling material by Dr. Torabinejad in 1995^19^. According to previous studies^20,21^, the antibacterial and antifungal properties of MTA were associated with elevated pH value. In the present study, direct contact test revealed antimicrobial effect of MTA against both *E. faecalis* and *P. gingivalis*. A previous study^2^ by Parirokh et al*.* showed that MTA exerts antibacterial effects against some facultative bacteria but not on any species of absolute anaerobes, however, another study by Kim et al*.*^22^ found that freshly mixed ProRoot MTA formed a bacterial growth inhibition zone against *P. gingivalis* in disk diffusion test. Since MTA has been tested in many researches but with contradictory results^20^ such difference may be attributed to the usage of different methodologies, bacterial strains, aerobic and anaerobic conditions.

Biodentine was developed as dentin replacement material. In addition to shorter setting time, it was also reported to be less porosity and less leakage^23^, less tooth discolors^24–26^ and excellent biocompatibility^27^ compared with MTA. In the present study, the antimicrobial effect of Biodentine against *E. faecalis* was similar to that of MTA, and the effect was lower when tested 7 days after setting, which is in accordance with a previous study by Koruyucu *et al.*^13^. Since the materials were kept in 100% humidity at 37 °C during the curing phase, one explanation could be that the antimicrobial components were continuously released along with the decrease of pH^3^.

iRoot FS (Innovative Bioceramix, Vancouver, BC, Canada) was introduced as a root canal repair material. As a premixed material, iRoot FS solidifies only when exposed to a moist environment. Previous studies have reported that iRoot FS has similar apical sealing ability and mechanical properties to MTA^28^ and that iRoot FS has a shorter setting time (initial 18 min and final 57 min) than MTA. There are great potentials for the clinical application of iRoot FS as the material is cytocompatible while facilitating cell adhesion, proliferation, differentiation and maintenance of normal cell function^29^. However, the antimicrobial effect of iRoot FS is unknown. In the present study, iRoot FS showed satisfactory antimicrobial effect when tested 20 min or 1 day after setting, and the effect became relatively lower than MTA and Biodentine when tested 7 days after setting, which might be attributed to its shorter setting time.

The pH values measured in this study were between 11 and 12, all the three materials showed strong alkaline pH, which is in accordance with previous studies^20,30^. However, though Biodentine exhibited the highest pH value at all time intervals, which might explain its superior antimicrobial effect 7 days after setting, it did not show the strongest antibacterial activity against *E. faecalis*. Therefore, as Zhang et al. mentioned in a previous study^31^, the antibacterial action cannot be rationally explained by pH alone. Moreover, in clinical situations, a desirable high pH after MTA application cannot be maintained due to the buffering capacity of dentin^22^.

The use of a single-species planktonic bacteria model is an evident limitation of our study. According to a previous study, the microbiota of persistent periapical infection is polymicrobial with predominance of *E. faecalis* and *P. gingivalis*, regardless of the method used for microbial identification^32^. Hence, *E. faecalis* and *P. gingivalis* were used in this study to appraise the antimicrobial property of these materials. However, in an infected root canal, a large number of microorganisms occur in parallel in the form of multispecies biofilms. Though simplified laboratory models do not represent the clinical reality of the infected root canal, they constitute valuable tools to preliminarily assess the antibacterial effect of endodontic materials, as they can be standardized and controlled^16^. This study provided a main insight into the antimicrobial effect of iRoot FS, and further studies against biofilms^33^ or in vivo studies are required to better understand the various properties of the retrograde filling materials.

## Conclusions

According to the findings of this study, fresh iRoot FS, Biodentine, and MTA killed *E. faecalis* and *P. gingivalis* effectively, but their antimicrobial effect decreased after 24 h, and distinctly decreased after 7 days after mixing. iRoot FS, Biodentine, and MTA showed a tendency of alkalinity during this 7-day experiment.

## Data Availability

The datasets generated during and/or analyzed during the current study are available from the corresponding author on reasonable request. All data generated or analyzed during this study are included in this published article.
